# Implementation of a portfolio assignment in the framework of an internship in dental higher education with a focus on preventive dentistry

**DOI:** 10.1186/s12909-026-09728-0

**Published:** 2026-06-19

**Authors:** Maria Hofmann, Niklas Krebs, Norbert Krämer

**Affiliations:** 1 Dental Clinic, Department of Paediatric Dentistry, Justus Liebig University Giessen, Schlangenzahl 14, Giessen, 35392 Germany; 2 Staff Division Studies, Teaching, Further Training and Quality Assurance (StL), Justus Liebig University Giessen, Giessen, Germany

**Keywords:** Higher education, Portfolio assignment, Reflection tasks, Preventive dentistry

## Abstract

**Background:**

Dental higher education requires teaching of critical thinking and reflective skills. Both of which are strengthened by portfolio assignments. The aim of this study was to evaluate how the implementation of a portfolio assignment in a preclinical course of dental studies affected the students‘ understanding of course topics and their motivation to self-reflect.

**Methods:**

Students in a preclinical course focusing on the topic of ‘preventive dentistry’ in the third semester of dental studies were given a newly developed portfolio assignment that they were to work on throughout the semester in relation to the internship and lecture content. At the end of the semester, the success of the portfolio assignment implementation was measured using a validated evaluation form. The form included two dichotomous items, 14 Likert scale items and two free text fields regarding “portfolio assignment in the context of dental studies”, “structure and tasks”, “content and knowledge gain”, “requirements and scope” and “overall assessment”.

**Results:**

Students of two consecutive semesters (n_total_ = 64), in which the portfolio was implemented for the first time, participated in the portfolio assignment and evaluation process. The evaluation results showed that portfolio assignments and self-reflection tasks have been underrepresented in dental studies to date. In both semesters, the portfolio assignment was rated as good to very good in > 90% of cases. The structure and tasks, as well as the repetition of course content, scope and effort, were rated positively. More than 80% of the students also agreed that the reflection tasks contributed to an increase in engagement with the course content and an awareness of the knowledge gained over the duration of the semester.

**Conclusion:**

In conclusion, the implementation of a portfolio assignment, including self-reflective elements, into the curriculum revealed to be a promising teaching tool that is well received by students. At the same time, portfolio assignments illustrate not only to students but also to teachers that learning and optimisation is a dynamic process that should be constantly evaluated and adapted in all semesters.

## Background

In undergraduate higher education in medicine and therefore also in the field of dentistry, curriculum requirements call for a critical, evidence-based approach to assessments and decisions [1–3]. A prerequisite for this is the ability of health professional students to engage in self-reflection [4, 5]. Reflective learning also has a positive impact on professional skills, such as empathy, which is particularly important in healthcare professions [6]. Portfolios in medical education are frequently used as a tool to promote reflective learning [7], which is an essential component of self-regulated learning (SRL). Portfolios typically contain tasks for (self-)reflection and are therefore suitable for practising the formulation of reflective texts at various levels of reflection [8] and there are few other tools that support and evaluate the learning competencies of students [9]. Portfolio assignments (PAs) further have the potential to improve students’ understanding and knowledge, linking theory and practice, increasing their knowledge, motivation and interest in specific themes and teaching them the ability to learn independently [8, 10–12]. They should therefore be seen as valuable tool in medical higher education. However, reflection exercises and PAs are not yet a regular part of the dental study curriculum.

The preclinical course ‘Internship in Dental Propaedeutics with a Focus on Preventive Dentistry’ (IDPPD) is a course that had to be newly established in accordance with the new regulations for the licensing of dentists in Germany, which have been valid since the winter semester of 2021/2022 [13]. At Justus Liebig University Giessen, this first dental course takes place in the third semester of the dentistry studies and is organised on an interdisciplinary basis covering three different disciplines (operative dentistry, periodontology and paediatric dentistry). All three disciplines deal with the primary prevention of oral diseases, such as dental caries, in everyday clinical practice. So far, the topic of dental prevention has been taught in the course through lectures, seminars and practical exercises.

Dental prevention for children and adult patients includes factors such as oral hygiene, non-cariogenic diet, fluoridation measures, regular visits to the dentist and individual saliva characteristics [14, 15]. Patient-specific dental prevention measures are also closely related to the determination of the patient’s individual caries risk [15]. The risk of caries can be determined using tools such as the “Cariogram” [16] or the “DAJ criteria” [17]. Both tools require information about the patient’s caries history. The caries history can be described using the DMFT value which refers to the number of decayed [D], missing [M] and filled [F] teeth [T] of a patient. All of the above factors are taught in theory and in practice as part of the IDPPD curriculum. Since competencies in oral health promotion of undergraduate dental students can be predicted by the reflective abilities gained in the context of PAs [18], portfolios seem to be a promising tool to specifically support the contents taught in the course.

In this study, a newly designed learning portfolio (a combination of a learning outcome portfolio and a learning process portfolio) was introduced and evaluated in the IDPPD course in two different cohorts (winter semester of 2024/2025 [WS] and summer semester of 2025 [SS]). The objectives of the PA were to improve the students’ acquisition of theoretical and practical course contents, the assessment of the students’ own caries risk based on the artefacts collected, and the documentation of the students’ learning progress. In addition, self-reflective exercises were implemented into the curriculum as part of the portfolio.

## Methods

### Aim and objectives portfolio assignment

The aim of this cross-sectional course evaluation study was to evaluate how the implementation of a PA in a preclinical course of dental studies affected the students’ understanding of course topics and their motivation to self-reflect. In accordance with the constructive alignment principle [19], the PA was created based on learning objectives that had been developed in advance. The following learning objectives were developed in accordance with the SMART (‘specific’, ‘measurable’, ‘attainable’, “relevant” and ‘time-bound’) criteria [20]:

After completing the theoretical and practical course elements and the accompanying PA, students should be able to …

- … determine their own caries risk and the caries risk of sample cases using various caries risk assessment strategies based on systems practised in the course or parameters they have collected themselves.
- …reflect on their knowledge of dental prevention and caries risk assessment at the beginning of the PA.
- …reflect on their own behaviour (oral hygiene, fluoride applications, dietary habits) against the background knowledge of dental prevention and caries risk assessment and give themselves advice to improve their own future preventive behaviour.
- …reflect on the extent to which knowledge has been gained and the extent to which a learning process has taken place, the extent to which the aspects learned are relevant to their future dental career, and which questions remain unanswered at the end of the portfolio (assignment).

The learning objective levels (according to Bloom [21]) to be addressed in the reflection exercises included in the portfolio are the cognitive-reflective and affective-reflective learning objective levels: the course content, the learning outcomes as well as the portfolio’s impacts and benefits for the future dental profession should be reflected upon.

The following learner activities [22] should also be actively promoted through the PA:

- Practice: Tasks for determining the DMFT value and for determining one’s own individual caries risk should be completed for practice and knowledge consolidation.
- Production: Students should collect their own data (various forms were to be filled out gradually in different sections of the course) in the clinical course which can then be used to determine their own caries risk using a variety of systems.
- Collaboration: In order to collect their own data, students are dependent on mutual support during the clinical exercises.
- Investigation: With the help of the information collected in the portfolio, students should be able to determine their own caries risk (and thus also that of other people for whom the same information is available) at the end of the semester.

### Contents and processing portfolio assignment

The selection of artefacts included in the portfolio and the scope of their processing were strictly specified. The portfolio was to be processed synchronously and asynchronously alongside the IDPPD and submitted shortly before the end of the semester after mutual clinical examinations and the subsequent analysis of the collected data, as well as after completing the reflection tasks. The specific tasks were completed individually by the students from the respective assignment until the portfolio was submitted. The PA was assessed as ‘passed’ or ‘failed’. No grades were given.

Structure and content of the portfolio:

- Initial reflection
- Main section
  - ◦ Exercises on DMFT values and DAJ criteria
  - ◦ Medical history form
  - ◦ Personal dental status report (recording of parameters from a collaborative clinical exercise)
  - ◦ Saliva test exercise (recording of parameters from a collaborative clinical exercise)
  - ◦ Exercise: dietary log, fluoride applications and personal oral hygiene, reflection on personal oral hygiene, dietary habits and recommendations
  - ◦ Exercise plaque index
  - ◦ Exercise: documentation of individual caries risk factors according to the Cariogram
  - ◦ Exercise: individual caries risk assessment according to the dental clinic’s own principle
- Final reflection

**Fig. 1:**
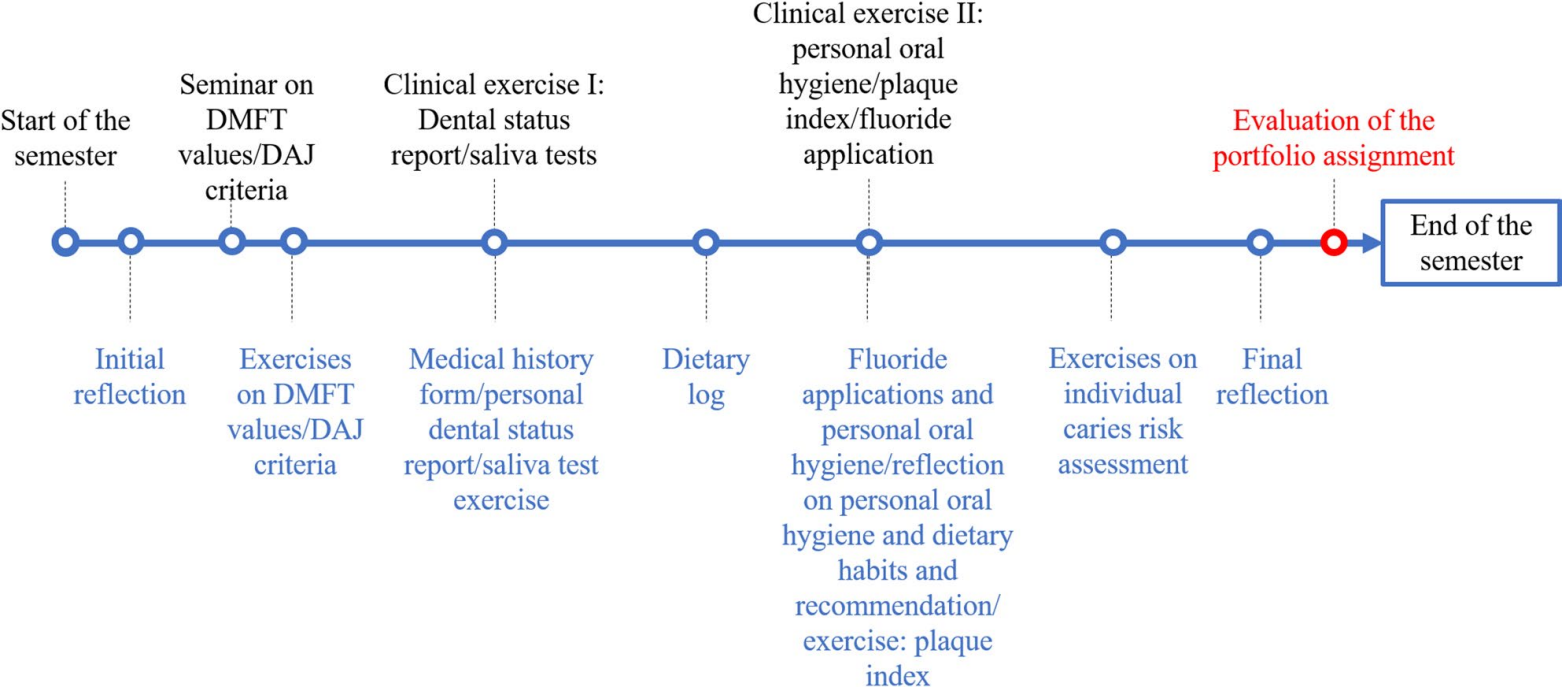
Tasks of the portfolio assignment (blue text) in relation to the course of the semester. Theoretical content was also covered in accompanying lectures. The timing of the evaluation is marked in red. DAJ = Deutsche Arbeitsgemeinschaft für Jugendzahnpflege [German Association for Youth Dental Care], DAJ criteria = criteria for determining patients’ caries risk, DMFT = sum of the decayed(D), missing (M) and filled (F) teeth, DMFT value = a value reflecting a patient’s history of caries

A diagram illustrating the individual steps of the PA in relation to the timeline of the semester can be found in Fig. 1.

### Reflection exercises

The reflection exercises (initial and final reflection) were a guided reflection processes in which the students were asked to reflect on their own learning process and knowledge gain with regard to the key topics using questions designed to foster reflective practice by means of complex competency tasks based on Bräuer [23] (‘describe, analyse, evaluate’).

The following questions were posed as part of the PA to guide reflective practice in form of complex competency tasks:

- Initial reflection task: ‘Reflect on your own prior knowledge of the topics “Dental prevention” and “Caries risk assessment”. Describe, analyse and evaluate what you already know. Which questions regarding the mentioned terms remain unanswered?’
- Final reflection task: “Please reflect on the following three questions (2 pages): Describe, analyse and evaluate how your knowledge of the topics ‘dental prevention’ and ‘caries risk assessment’ has changed. Which questions remain unanswered? What is still unclear? To what extent are the aspects you have learned relevant to your future dental practice?”

### Assessment tool: evaluation form

To retrospectively evaluate the PA at the end of each semester, an evaluation form was newly developed in collaboration with the Network for Teaching Learning Development Mittelhessen (https://www.hd-mittelhessen.de/wp/hdm/start-hdm/).

The primary evaluation outcomes were: the perceived learning benefit, the reflective engagement, the perceived professional relevance and the acceptance/satisfaction from the students’ perspective.

The evaluation form contained 18 items (including two dichotomous items, 14 Likert scale items and two free text fields). The items related to the following five topics: ‘PA in the context of dental studies’, ‘Structure and tasks’, ‘Content and knowledge gain’, ‘Requirements and scope’ and ‘Overall assessment’.

The evaluation results of the dichotomous and the Likert scale items were analysed using SPSS software (IBM SPSS Statistics version 29.0.2.0, IBM Corp., Armonk, USA, https://www.ibm.com/spss). A Mann-Whitney U test was conducted to assess the two different semesters for significant differences in the responses to these items. Cronbach’s alpha was also calculated to assess the reliability of the Likert scale items. The most important points of the free text items were summarised descriptively.

This study involved the analysis of data from voluntary student course evaluations. The anonymity of the evaluation was ensured by the number of participants (> 5 people), the decision not to collect demographic data, and the separate analysis of the Likert scale and free text items. To avoid response bias resulting from skewed results due to non-participation in the evaluation, the evaluation was conducted on a voluntary basis during the usual teaching hours. The Ethics Committee of Department 11 (Medicine) at Justus Liebig University Giessen waived the requirement for informed consent due to the anonymous and non-invasive nature of the evaluation and the use of data collected as part of routine educational procedures.

## Results

In the WS, 33 students took part in the evaluation, and in the SS, 31 students took part, meaning that all course participants (100.0%) in both semesters took part in the evaluation.

The results of the analysis of the evaluation forms for the WS and the SS for the Likert scale categories ‘PA in the context of dental studies’, ‘Structure and tasks’, ‘Content and knowledge gain’ and ‘Requirements and scope’ can be found in Table 1. The reliability check of the 14 Likert scale items yielded a Cronbach’s alpha value of 0.776 for the 14 Likert scale items.

**Table 1:** Presentation of the results for the Likert scale items in the evaluation questionnaire for the categories ‘Structure and tasks’ and ‘Content and knowledge gain’

Item		Strongly agree.	Somewhat agree.	Neither agree nor disagree.	Somewhat disagree.	Strongly disagree.
Structure and tasks						
The tasks in the portfolio were arranged in a logical order.	WS	24 (72.7)	9 (27.3)	0 (0.0)	0 (0.0)	0 (0.0)
	SS	21 (67.7)	10 (32.3)	0 (0.0)	0 (0.0)	0 (0.0)
The tasks in the portfolio were clearly formulated.*	WS	21 (63.6)	12 (36.4)	0 (0.0)	0 (0.0)	0 (0.0)
	SS	11 (35.5)	20 (64.5)	0 (0.0)	0 (0.0)	0 (0.0)
The tasks in the portfolio established cross-references to topics covered in the course or lecture.	WS	25 (75.8)	6 (18.2)	1 (3.0)	1 (3.0)	0 (0.0)
	SS	25 (80.6)	6 (19.4)	0 (0.0)	0 (0.0)	0 (0.0)
Content and knowledge gain						
The portfolio work has helped to strengthen my knowledge and understanding of the subject of ‘preventive dentistry/dental prophylaxis’.	WS	16 (48.5)	14 (42.4)	2 (6.1)	1 (3.0)	0 (0.0)
	SS	13 (41.9)	15 (48.4)	2 (6.5)	1 (3.2)	0 (0.0)
The portfolio work has helped to strengthen my knowledge and understanding of the subject area of ‘caries risk assessment’.	WS	20 (60.6)	13 (39.4)	0 (0.0)	0 (0.0)	0 (0.0)
	SS	20 (64.5)	11 (35.5)	0 (0.0)	0 (0.0)	0 (0.0)
Working on the portfolio has made me feel better prepared for the course exam.	WS	2 (6.1)	15 (45.5)	11 (33.3)	5 (15.2)	0 (0.0)
	SS	5 (16.1)	17 (54.8)	6 (19.4)	3 (9.7)	0 (0.0)
The portfolio work has helped to improve my future work in the dental profession.	WS	5 (15.2)	20 (60.6)	6 (18.2)	2 (6.1)	0 (0.0)
	SS	10 (32.3)	13 (41.9)	6 (19.4)	2 (6.5)	0 (0.0)
The reflection tasks as part of the portfolio work encouraged me explore the topics of ‘dental prophylaxis’ and ‘caries risk assessment’.	WS	14 (42.4)	13 (39.4)	2 (6.1)	3 (9.1)	1 (3.0)
	SS	15 (48.4)	12 (38.7)	2 (6.5)	1 (3.2)	1 (3.2)
Reflecting on my progress at the beginning and end of the portfolio work helped me to consciously perceive my increase in knowledge over the course of the semester.	WS	15 (45.5)	14 (42.4)	1 (3.0)	2 (6.1)	1 (3.0)
	SS	18 (58.1)	10 (32.3)	3 (9.7)	0 (0.0)	0 (0.0)
I consider the portfolio to be a useful support for the dental medicine course curriculum in the third semester.	WS	7 (21.2)	23 (69.7)	2 (6.1)	1 (3.0)	0 (0.0)
	SS	14 (45.2)	14 (45.2)	3 (9.7)	0 (0.0)	0 (0.0)

The results are presented as absolute and relative (in relation to the size of the semester) frequencies [n (%)].WS = 2024/2025 winter semester. SS = 2025 summer semester. Number of students in WS = 33. Number of students in SS = 31

*= significant differences in the responses to this item between WS and SS (Mann-Whitney U test, *p* = 0.026)

With regard to the category ‘PA in the context of dental studies’, 97.0% of students in the WS and 100.0% of students in the SS stated that they had never created a PA for any other course in their dental studies. Furthermore, 81.8% of students in the WS and 96.8% of students in the SS stated that they had not yet worked on assignments involving elements of self-reflection in other courses during their dental studies. The evaluation results for categories ‘Structure and tasks’ and ‘Content and knowledge gain’ are presented in Table 1.

The following results were obtained in the ‘Requirements and scope’ category: In the WS, 90.9% (SS: 87.1%) of students considered the requirements to be ‘just right’, while 9.1% (SS: 12.9%) considered them to be ‘slightly too low’. In the WS, the scope of the PA was rated as ‘just right’ by 93.9% (SS: 96.8%), as ‘slightly too low’ by 3.0% (SS: 3.2%) and as ‘slightly too high’ by 3.0%. Each week, 36.4% of students in the WS spent one hour working on the PA (SS: 25.8%), 57.6% (SS: 71.0%) stated that they spent less than one hour per week on the PA, and 6.1% (SS: 3.2%) stated that they spent two hours per week on the PA.

For the category ‘Overall assessment’, 24.2% of the portfolio was graded ‘very good’, 69.7% ‘good’ and 6.1% ‘satisfactory’ in the WS. In the SS, 38.7% of students rated the portfolio as ‘very good’, 54.8% as ‘good’, 3.2% as ‘satisfactory’ and 3.2% abstained.

The evaluation of the free text item ‘What I particularly liked about the PA was…’ showed that the reflection exercises in the portfolio were particularly well received. Students appreciated becoming aware of their own knowledge growth by comparing reflections at the beginning and end of the semester, which elicited positive feelings. The connection between the content and timing of the PA and the course content as well as theoretical lecture content were rated particularly positively. The portfolio lead students to repeat and consolidate their knowledge, which also contributed to exam preparation. The practical collection of their own oral hygiene and health data was also highly appreciated, as it promoted awareness of their own oral health and illustrated the course content. Elements such as medical history forms, dietary logs, caries risk assessment and saliva tests were found to be particularly interesting. According to the results of the evaluation, the PA showed a practical relevance in terms of dental prophylaxis, which, from the students’ perspective, facilitated the transfer of learning content to later dental practice and created a better understanding of patient perspectives. The collaboration with peers and the playful nature of the tasks were highlighted positively. The structure of the portfolio, which offered a clear, systematic plan throughout the semester, and the flexible processing time against the background of its implementation in the overall timetable were also praised.

The free text item ‘I see room for improvement in the project work regarding the following aspects…’ was evaluated as follows. The students wanted improvements to the portfolio, especially the layout, as some pages were considered to be confusing or printed too small; the exercises relating to the dietary log were mentioned in particular. Extension references to course and lecture content, including from other departments, was requested. A few students criticised the reflection tasks as too vague and not very useful; some of them wished for multiple-choice alternatives. Additional quiz tasks similar to the end-of-semester exam were requested. In terms of organisation, a folder was suggested to make the portfolio easier to handle, and the submission deadline was proposed to be moved to regular lecture days. The students wanted to keep the portfolio for exam preparation and not have to submit it in advance. Some students felt that filling out their own medical history form was unnecessary. In addition, students wished for more tasks on topics such as DMFT values, primary teeth, dental anatomy and exemplary clinically relevant case exercises. A greater amount of teamwork exercises and more detailed feedback as well as the return of the assessed portfolio were also requested. In addition, a discussion of the portfolio results in the course was considered useful. The students further stated that the scope of the PA should not be expanded in order to ensure compatibility with other subjects and exams.

## Discussion/conclusions

### Methodology discussion

In this study, an analogue portfolio with a limited number of pages was introduced and evaluated. Another option would have been to implement an electronic portfolio (e-portfolio), which would have facilitated access to portfolio documents from multiple locations. In a study by Song et al. (2024), medical students in their clinical training phase rated e-portfolios as more helpful than paper portfolios in terms of providing a means of continuous documentation and tracking learning progress during clinical practice [24]. However, the implementation of an e-portfolio is even more complex and time-consuming due to technical constraints and not only in terms of training [24–26]. Ensuring data security also remains a challenge when using e-portfolios. This aspect is particularly critical in light of the documentation of genuine, student-owned oral health-related data. At the same time, an analogue portfolio offers advantages in terms of training dexterity, which is beneficial in the context of vocational training in dental professions. In addition, an analogue portfolio is intended to prevent the extension of the portfolio content and the misuse of artificial intelligence. Nevertheless, the switch to an e-portfolio may be implemented in future courses once all the necessary infrastructure is in place.

Since personal data (findings on one’s own dental status, oral hygiene, diet, saliva characteristics, etc.) are processed as part of the portfolio, only teachers were permitted to view the portfolios. There was no detailed grading, as the main aim was for students to understand, based on their own personal examples, how dental prevention and the assessment of individual caries risk work and why they are important in dental practice. However, the complete absence of assessment carries the risk that the PA is not taken seriously enough especially since students are very busy with other subjects in the third semester.

The internal consistency of the items in the newly developed evaluation questionnaire can be classified as acceptable with a Cronbach’s alpha of 0.77. Nevertheless, the small number of evaluations (*n* = 64) represents a limitation of the study, which may be acceptable for a preliminary educational evaluation, but the results should be verified by further studies with larger sample sizes, e.g. in a multicentre format. The monocentric design of the study is also a limitation. However, it should be mentioned at this point that the structure of the IDPPD course as described varies from university to university. Since new regulations for the licensing of dentists have only been in place for a short time in Germany, other dental institutions may wish to adopt the promising PA concept in the context of preclinical preventive dentistry courses in the future. Nevertheless, a multicentre study design would enhance the generalisability of the results, particularly given the heterogeneous study groups and conditions at other sites. Furthermore, this could foster cooperation between the participating universities and promote the standardisation of teaching content.

### Discussion of portfolio evaluation results

The full participation of students in the evaluation of the PA can be explained by the fact that the evaluation was integrated into the course time of the IDPPD. This could have led to distortions in the results. For example, respondents may have tended to give positive answers because the lecturers were present in the same room, or participants may have felt subject to peer pressure. Nevertheless, the students did not have to make any additional effort outside of course hours, and it was not only particularly dissatisfied students or particularly motivated students who took part. To give students a sense of security, the anonymity of the data collection was emphasised. The lecturer present during the evaluation spent the evaluation period preparing the materials for the practical part of the course, meaning that students were not observed whilst completing the questionnaire. The profiles of the evaluation results were very similar in both semesters (see Table 1), which indicates that the portfolio is highly reproducible and reusable in heterogenous groups of learners. This is supported by the fact that the two semesters examined differed significantly only in terms of the response to a single item (item 4, see Table 1) and also only in terms of a shift in one value on the Likert scale (“strongly agree” vs. “somewhat agree”).The particularly positive ratings given to the PA by almost all participating students suggest a high level of acceptance; however, this observed ceiling effect limits the variance in the responses. This should be regarded as a limitation when interpreting the results and could be avoided in future by using a more refined scale or items formulated in even more specific terms in the evaluation questionnaire.

Compared with medical education, portfolios have so far rarely been used in dental education, a finding confirmed by the evaluation results of this study (see Table 1). The results showed that for the absolute majority of students of both semesters, PAs and self-reflection tasks were completely new in the context of dental studies, which emphasizes the need to implement such teaching tools. Particularly in the pre-clinical phase of the dental curriculum, there is little evidence regarding the use of portfolio-based learning in higher education. Following a five-year implementation phase of portfolio-based assessment in clinical dentistry, Gadbury-Amyot and Overman (2018) describe how portfolios in dental education enable a holistic, continuous and reliable assessment of competencies. At the same time, however, portfolio-based assessment in this context also requires considerable preparation and support, particularly with regard to the ability to reflect [27].

Slepcevic-Zach and Stock (2018) as well as Dailey (1998) describe how creating a portfolio is itself a process of integrating theory and practice. According to both papers, a portfolio provides a structured framework for reflection and selection of evidence, thereby promoting the connection between theoretical knowledge and practical application. In addition, the portfolio makes it possible to visualise development and learning over time [28, 29]. Both effects were reported by the students in this study: the students reported that they noticed cross-references to course and lecture content. The evaluation results also showed that students consciously perceived the structure of the PA and the link between the PA and the theoretical as well as practical course tasks.

The results of the Likert scale and the free text items showed that the students rated the PA as successful in deepening their learning, particularly with regard to the key topics of “preventive dentistry” and “caries risk assessment”. This is consistent with findings from the literature, which also describe how PAs help students gain awareness of the objectives they have to achieve [12].

From the students’ perspective in this study, the PA was considered somewhat less effective, but still good and satisfactory, as preparation for the course examination and for the future dental profession. This could be due to the fact that students in the IDPPD course are encountering dental content for the first time in their entire course of study and do not yet have a clear idea of its clinical application. In addition, the exam was only written after the PA had been evaluated, which could explain the uncertainty in assessing its usefulness for exam preparation.

The majority of the students also agreed that the reflection tasks contributed to an increase in engagement with the course content and an awareness of the knowledge gained over the duration of the semester. An analysis of data from medical training by van der Gulden et al. (2020) shows that the intended effects of the SRL are only visible or documented to a limited extent [30]. This, in turn, makes it difficult to measure the impact of implementing a PA.

With regard to the time required to complete the PA, most students stated that they spent an average of less than one hour to one hour per week on the PA. The requirements and scope of the PA were rated as ‘just right’ by the majority of the students. In contrast to the results of this study, many students in other studies consider the time intensiveness of PA to be a major disadvantage [26, 31]. In the present study, the time required seems to have been well estimated. The compatibility with the general study schedule for the third semester was also praised by students in the free text fields. From the students’ perspective, the scope of the PAs should not be expanded significantly in order to avoid time conflicts, especially during the examination periods at the end of the semester.

The PA was rated good to very good on average in both semesters which indicates that the implementation of the PA was perceived as successful by the students. The students also agreed that the PA was a useful addition to the curriculum of the IDPPD course.

The students also appreciated the collaborative and “playful” nature of many of the tasks of the portfolio. These description of the exercises as having a “playful” character suggests that the students did not simply work through the tasks of the PA but also enjoyed and appreciated them. Other authors also describe how PAs are considered enjoyable and valuable by students [32, 33].

The students also described their perception of their knowledge growth over the duration of the semester through working on the reflection tasks. They furthermore stated that they appreciated being “forced” to repeat the course content through the tasks in the portfolio, which indicates that working on the PA deepened the students’ knowledge. These benefits are also described in other publications on PAs in dental studies [34, 35].

The evaluation results suggested that the PA increased the students’ self-awareness and motivation with regard to course content as they perceive the character of the tasks of the portfolio as “playful”.

The evaluation of the PA also indicated that students became more aware of their own learning progress. The cooperative course design with the two other disciplines, operative dentistry and periodontology, will continue and may even be extended to the portfolio.

Students further reported in the free text fields that they received a better understanding of patients’ perspective by working on the tasks of the portfolio that were related to their personal oral health data. This leads to the suggestion that these kinds of tasks are able to make the students more empathetic which is one of the core competences in medical professions [36]. To emphasise the importance of the learning experience on a psychomotor and an empathic level, the future tasks in the portfolio could be supplemented with the following additional reflection questions: “How did you feel about determining your own caries risk and collecting the necessary parameters (general medical history, dental status findings, nutritional and fluoride history, saliva tests, etc.)? What conclusions do you draw from this for dealing with future patients or for advising future patients on caries risk assessment and dental prevention?”.

The suggestions for improvement in the PA were entirely constructive in nature which, incidentally, also speaks for a good relationship between the teachers and students. The literature describes how PAs lead to a shift in the distribution of roles between students and teachers [25, 26]. This is supported by the present study: students can work on tasks independently and flexibly in terms of time, while teachers no longer have to model every single step and immediately evaluate or correct it. This is beneficial for both sides and creates trust in the other party. It can also promote students’ sense of self-awareness. Awareness of one’s own competence is described as an essential force for personal development [37]. This is further emphasised by the results of López-Crespo et al., which showed that students’ self-efficacy is also increased by working on self-reflection tasks [38]. At the same time, the students’ reflective texts can promote the teachers’ awareness of students’ needs [8, 10] and help students recognize their learning needs [10]. There were even requests to include additional course topics in the portfolio or additional learning content and exercises, which would mean extra work for the students and highlights the positive reception of the portfolio by the students. This observation, the fact that portfolios increase the students’ intrinsic motivation, is also shared by Ciesielkiewicz (2019), who concludes that students view PAs not only as part of their coursework for the purpose of obtaining a grade but also as a learning tool [39].

A few students did not feel like the reflection tasks were beneficial to them. Detailed instructions are recommended as a guide, especially for students with no previous experience of reflection [5]. In this case, one possible strategy would be to carry out a brief evaluation of how to deal with or access the reflection tasks after the initial reflection task and, if necessary, to provide further instructions on an individual basis. In addition, students could be made aware that these reflection tasks are intended to facilitate self-reflection: a shift from non-responsibility to responsibility. The reflection process therefore involves more than just describing a successful solution strategy. One of the achievements of reflection exercises is being able to express a problem, even if the problem seems unsolvable [40].

In both semesters, new ideas for optimising the PA emerged. This suggests that not only students should be accustomed to a dynamic learning process but that teachers should also continue to adapt and to optimise their teaching. In particular, opportunities for teachers to be available for consultation should be established in the form of consultation hours for more intensive coaching.

Furthermore, with regard to the assessment of the PA, students would have liked to receive more feedback from the assessing lecturers. A dialogue on portfolio assessment is planned for future semesters.

A further limitation of the study is the fact that the evaluation results consist primarily of self-reported student perceptions. Therefore, when interpreting the results, it should be noted that these are subjective statements which do not automatically allow conclusions to be drawn regarding an objective improvement in learning outcomes. Whilst this may be appropriate for an initial implementation study, it should also be examined in further studies using objective measurement methods, e.g. through additional standardised performance tests.

### Conclusion on the implementation of a portfolio assignment

Taken the limitations into account, it can be concluded that the implementation of a PA, including self-reflective elements, into the curriculum of a practical course dealing with “preventive dentistry” is a promising teaching tool, which is subjectively well received by students and can be successful with good instruction. At the same time, PAs illustrate not only to students but also to teachers that learning and optimisation is a dynamic process that should be constantly evaluated and adapted over all subsequent semesters. The concept of portfolio-based learning may also have implications for higher education in other medical disciplines, where the ability to reflect upon and deepen the understanding of subject matters taught needs to be enhanced.

## Data Availability

The data supporting the findings of this study are not publicly available due to confidentiality and data protection concerns related to student evaluations. The data are archived at the Department of Paediatric Dentistry, Justus Liebig University Giessen, Giessen, Germany, and may be made available upon reasonable request to the corresponding author.

## Abbreviations

DAJ: Deutsche Arbeitsgemeinschaft für Jugendzahnpflege [German Association for Youth Dental Care]
DMFT: Sum of the decayed (D), missing (M) and filled (F) teeth
IDPPD: Internship in Dental Propaedeutics with a Focus on Preventive Dentistry
PA: Portfolio assignment
SRL: Self-regulated learning
SS: 2025 summer semester
WS: 2024/2025 winter semester
